# Comparison of Platelet-Rich Plasma and Corticosteroid Injections for Chronic Plantar Fasciitis: A Randomized Controlled Trial

**DOI:** 10.7759/cureus.59656

**Published:** 2024-05-04

**Authors:** Krishan Kumar, Vikas Rao, Amrit Panda, Sathyendra K.G., Harshvardhan Buddhist

**Affiliations:** 1 Orthopaedics, Sports Injury Centre, Vardhman Mahavir Medical College (VMMC) & Safdarjung Hospital, New Delhi, IND; 2 Orthopaedics, Employees State Insurance-Post Graduate Institute of Medical Sciences and Research (ESI-PGIMSR), New Delhi, IND

**Keywords:** chronic heel pain, plantar heel pain, corticosteroid, platelet-rich plasma, plantar fasciitis

## Abstract

Background: Plantar fasciitis is a common and debilitating foot condition, with varying treatment options and inconsistent outcomes. The objective of this study was to assess and compare the effectiveness of autologous platelet-rich plasma (PRP) injections and corticosteroid injections in treating persistent plantar fasciitis.

Methods: In this study, a total of 70 patients suffering from chronic plantar fasciitis were randomly divided into two groups, i.e., one receiving PRP injections (n=35) and the other receiving corticosteroid injections (n=35). The visual analog scale (VAS) was used to assess pain outcomes, while the American Orthopaedic Foot and Ankle Society (AOFAS) score was used to assess functional status. Patients were assessed before the injection and then followed up at 15 days, one month, three months, and six months after the injection.

Results: The baseline VAS and AOFAS scores were similar between the two groups. However, the PRP group showed significantly greater improvements in VAS and AOFAS scores compared to the corticosteroid group at the one-month, three-month, and six-month follow-ups (p<0.05). The PRP group had a higher proportion of patients with mild or moderate pain and better functional outcomes at later time points.

Conclusions: Autologous PRP injections are superior to corticosteroid injections in terms of long-term pain alleviation and functional improvement for patients suffering from chronic plantar fasciitis. Platelet-rich plasma should be regarded as a feasible therapeutic choice for this condition, especially in individuals who have not shown improvement with conservative treatment.

## Introduction

Plantar fasciitis is a prevalent foot and ankle condition that presents with a gradual onset of heel pain that worsens with the initial few steps after a period of rest [1,2]. The etiology is multifactorial, and risk factors include obesity, decreased ankle dorsiflexion, and prolonged weight-bearing activities [3,4]. While the exact pathogenesis is not fully understood, it is thought to involve a degenerative process rather than pure inflammation [5,6].

Despite the availability of various treatment modalities, the management of plantar fasciitis remains challenging, with inconsistent outcomes and a lack of consensus on the optimal treatment approach [7,8]. Non-steroidal anti-inflammatory medications (NSAIDs), corticosteroid injections, stretching exercises, and orthotics are examples of commonly used interventions [9,10]. However, the use of corticosteroids has been associated with potential complications, such as rupture of the plantar fascia and atrophy of the fat pad [11].

In recent years, the use of autologous platelet-rich plasma (PRP) has gained attention as an alternative treatment for chronic tendinopathies, including plantar fasciitis [12-15]. Platelet-rich plasma is a blood-derived product with a higher concentration of growth factors, which may stimulate tissue regeneration and healing [16,17]. While some studies have reported favorable outcomes with PRP injections in plantar fasciitis, the comparative efficacy of corticosteroids remains unclear [18-23]. This study aimed to compare the clinical effectiveness of PRP versus corticosteroid injections in the management of chronic plantar fasciitis.

## Materials and methods

Study design and participants

This was a prospective randomized controlled trial conducted in the Department of Orthopaedics at a tertiary care hospital from January 2019 to December 2019. The trial was registered in the Clinical Trial Registry-India (CTRI) (No. CTRI/2019/01/016952). Institutional Ethics Committee clearance was obtained from the Employees State Insurance-Post Graduate Institute of Medical Sciences & Research (ESI-PGIMSR) for our study. A patient diagnosed with plantar fasciitis was first treated conservatively for the first three months using physiotherapy, splints, and NSAIDs. Physiotherapy included plantar fascia strengthening and stretching exercises, along with stretching of the calf muscle. Patients who did not respond to the conservative treatment were included in the study.

Inclusion and exclusion criteria

Patients diagnosed with plantar fasciitis, patients faced with failure of conservative treatment for at least three months, and patients aged between 20 and 60 years were included in the study. Patients with any previous local injection treatment for heel pain, a history of surgery for heel pain, associated pathology involving the foot and ankle (diabetic ulcers, charcot foot, osteomyelitis), and systemic disorders such as uncontrolled diabetes mellitus, rheumatoid arthritis, hematological disease, or gout were excluded from the study.

After obtaining informed consent, eligible patients were randomly allocated to receive either PRP (n=35) or corticosteroid (n=35) injections. This randomization was done using computer-generated random numbers. A statistician generated a list of random numbers and allocated them to the two treatment groups (steroid and PRP). These allocated random numbers were then placed inside separate sealed envelopes by the statistician. A data enumerator opened the sealed envelopes consecutively, one by one, without any exceptions. The information inside each envelope determined whether the participant would be assigned to the steroid group or the PRP group. Before allocating a participant to a treatment group, their eligibility was confirmed. Once confirmed, the data enumerator opened the next envelope in the sequence and recorded the treatment allocation (steroid or PRP) on a randomization list. Two groups, i.e., group A and group B of 35 people each with similar baseline characteristics, were formed. Group A received local autologous PRP injections, while Group B received local corticosteroid injections.

Intervention procedures

For the PRP group, 20 mL of venous blood was collected and processed using a standardized two-step centrifugation protocol to obtain 3 mL of PRP. Each vacutainer contained acid-citrate dextrose (ACD). In the first spin, the vacutainers were centrifuged at 1300 rpm for 10 minutes. The blood was separated into three layers after the first spin. The bottom layer comprised RBCs, the middle layer was the buffy coat (leucocytes with platelets), and the top layer was plasma with suspended platelets. The serum and the buffy coat (leucocytes and platelets) were drawn from each vacutainer into another sterile test tube. 

For the second spin, the test tube was returned to the centrifuge machine and was centrifuged again at 3500 rpm for 10 minutes. After the second spin, the test tube contained platelet-poor plasma on top and platelets and leucocytes at the bottom. The supernatant was drawn and discarded, leaving a platelet-rich fraction of plasma at the bottom [24].

Under strict aseptic conditions, the PRP was injected into the most tender point of the plantar fascia using a peppering technique. The corticosteroid group received a 1 mL (40 mg) injection of methylprednisolone mixed with 2 mL of 2% lidocaine, administered similarly.

Post-procedure protocol

Immediately after injection, the patients were observed for 15 to 20 minutes. They were advised to apply ice on the injected area for swelling and pain control and to avoid high-impact activities for a week. All the patients were taught stretching exercises for the plantar fascia and Achilles tendon. Patients were allowed to take paracetamol for pain after the injection.

Outcome measures

The primary outcome measures were the visual analog scale (VAS) for pain and the American Orthopaedic Foot and Ankle Society (AOFAS) score for functional status. Assessments were performed before the injection and then followed up at 15 days, one month, three months, and six months post-injection.

Statistical analysis

**Figure 1:**
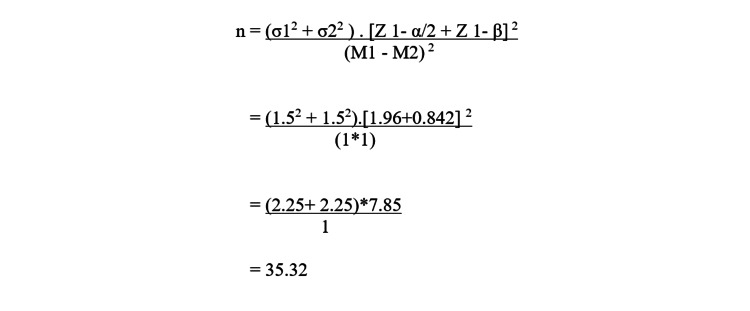
Formula for calculating sample size Where Zα/2 is the critical value of the normal distribution at α/2 (for a confidence level of 95%, α is 0.05 and the critical value is 1.96), Zβ is the critical value of the normal distribution at β (for a power of 80%, β is 0.2 and its critical value is 1.282) and σ 1 and σ 2 are the standard deviations of the two groups and M1 and M2 are the means of two groups.

Our estimated sample size was based on efficacy in terms of VAS in two groups. The minimally clinically significant difference in VAS between the two groups was considered to be 1. To detect a difference of 1 assuming a standard deviation of 1.5, using a two-sided alpha of 0.05 and power of 80% while accounting for 10% loss to follow-up, 35 patients were included in each study arm. The formula for the calculated sample size is shown in Figure 1.

Statistical testing was conducted with the SPSS Statistics version 17.0 (IBM Corp., Armonk, NY, USA). Continuous variables were presented as mean±SD or median if the data was unevenly distributed. Categorical variables were expressed as frequencies and percentages. The comparison of continuous variables between the groups was performed using Student’s t-test. Nominal categorical data between the groups were compared using the chi-squared test or Fisher’s exact test as appropriate. Non-normal distribution continuous variables were compared using Mann Whitney U test. For all statistical tests, a p-value less than 0.05 was taken to indicate a significant difference.

## Results

A total of 70 patients were included in the analysis, with 35 patients in each group. The mean age of the study population was 40.46 ± 8.5 years, and 57.14% were male. The most common age group affected with plantar fasciitis were those between the ages of 31 to 40 years and 41 to 50 years.

The VAS score for pain

**Figure 2:**
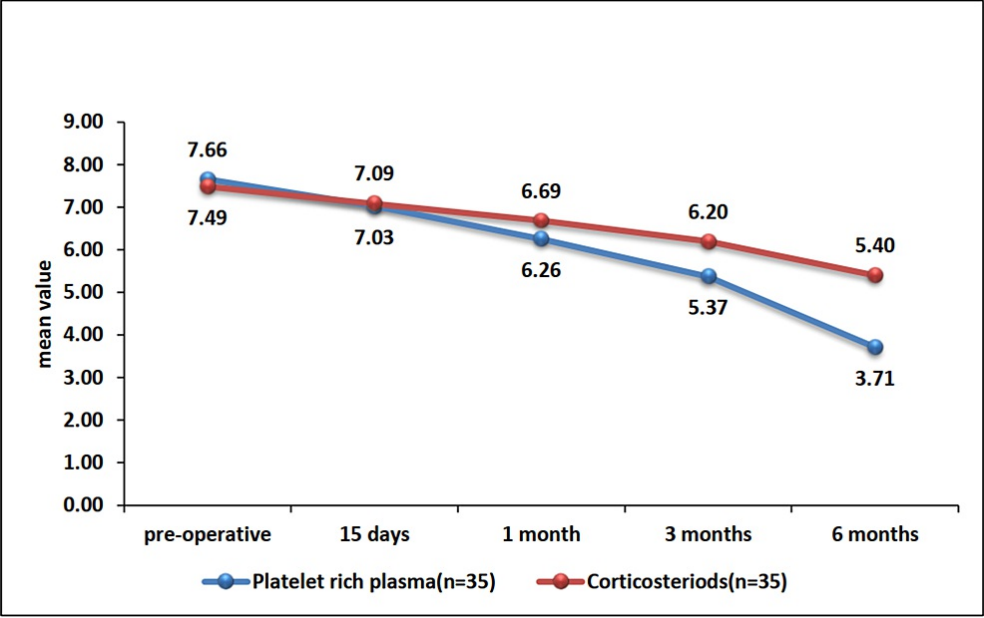
Comparison of the trend of VAS scores at different time intervals between the PRP and corticosteroid groups VAS: Visual analog scale

The baseline mean VAS scores were similar between the PRP and corticosteroid groups (7.66 ± 1.0 vs. 7.49 ± 1.2, p=0.608). However, the PRP group showed significantly greater improvements in VAS scores compared to the corticosteroid group at the one-month (6.26 ± 0.7 vs. 6.69 ± 0.87, p=0.035), three-month (5.37 ± 0.69 vs. 6.2 ± 0.72, p<0.0001), and six-month (3.71 ± 0.67 vs. 5.4 ± 0.65, p<0.0001) follow-up (Table 1, Figure 2).

**Table 1:** Comparison of VAS score and AOFAS score at baseline, 15 days, one month, three months, and six months between the PRP and corticosteroid groups ^+^Statistically significant at p < 0.05 VAS: Visual analog scale, AOFAS: American Orthopaedic Foot and Ankle Society

Variables	PRP-treat­ed group (n=35)	Corticosteroid-treated group (n=35)	p-value^+^
	Mean ± SD	Mean ± SD	
VAS score			
At baseline	7.66 ± 1	7.49 ± 1.2	0.608
At 15 days	7.03 ± 0.79	7.09 ± 0.98	0.883
At 1 month	6.26 ± 0.7	6.69 ± 0.87	0.035
At 3 months	5.37 ± 0.69	6.2 ± 0.72	<0.001
At 6 months	3.71 ± 0.67	5.4 ± 0.65	<0.001
AOFAS score			
At baseline	62.86 ± 7.1	64 ± 8.12	0.527
At 15 days	65.71 ± 9.17	65.14 ± 8.87	0.788
At 1 month	72.57 ± 9.8	68 ± 9.94	0.059
At 3 months	79.43 ± 3.38	72.57 ± 9.8	0.001
At 6 months	84 ± 4.97	79.43 ± 3.38	<0.001

The AOFAS score

**Figure 3:**
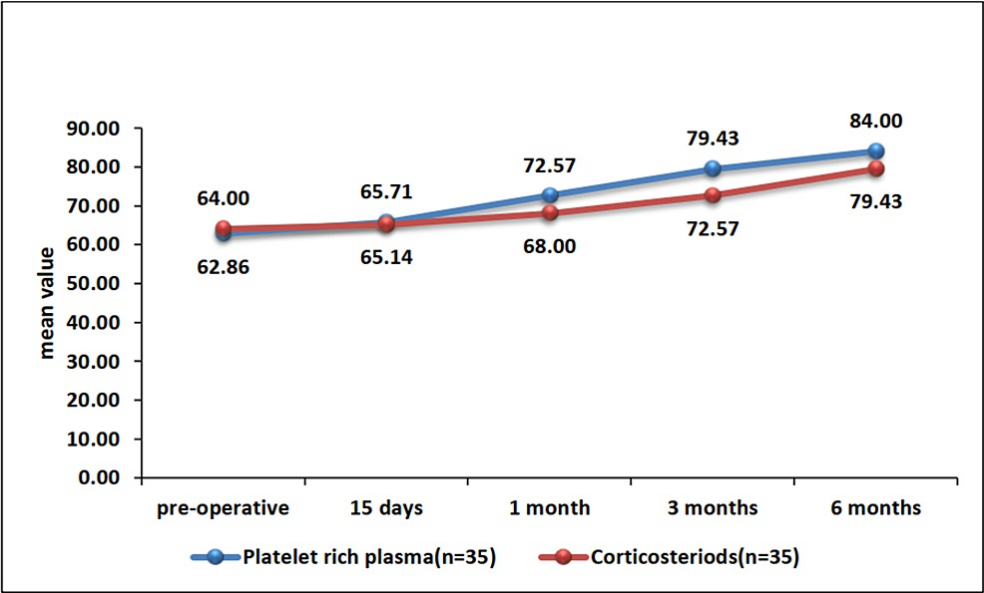
Comparison of the trend of AOFAS scores at different time intervals between the PRP and corticosteroid groups AOFAS: American Orthopaedic Foot and Ankle Society

The baseline AOFAS scores were comparable between the two groups (62.86 ± 7.1 vs. 64 ± 8.12, p=0.527). At the one-month (72.57 ± 9.8 vs. 68 ± 9.94, p=0.059), three-month (79.43 ± 3.38 vs. 72.57 ± 9.8, p=0.001), and six-month (84 ± 4.97 vs. 79.43 ± 3.38, p=0.0002) follow-up, the PRP group had significantly higher AOFAS scores compared to the corticosteroid group (Table 1, Figure 3). None of the patients reported any adverse effects post the injections.

## Discussion

The study found that the most common age groups affected by plantar fasciitis were those between 31 to 40 years and 41 to 50 years, which is consistent with the typical age range for this condition. The study population had a higher proportion of males compared to females, which could be due to occupational or lifestyle factors that put males at a higher risk for plantar fasciitis. The findings are comparable with other studies (Klein et al. [25] and Goweda et al. [26]) that also reported a higher prevalence of plantar fasciitis in middle-aged individuals and a slightly higher incidence in males.

The present study demonstrates that local injection of autologous PRP is more effective than corticosteroid injection in providing long-term pain relief and functional improvement in patients with chronic plantar fasciitis. The PRP group showed significantly better outcomes in terms of VAS scores and AOFAS scores at the one-month, three-month, and six-month follow-ups.

The superior efficacy of PRP may be attributed to its ability to deliver a higher concentration of growth factors, which can stimulate tissue regeneration and healing. Platelet-rich plasma contains a high concentration of growth factors and other bioactive proteins released from the patient's platelets. These factors, such as platelet-derived growth factor, transforming growth factor beta, and vascular endothelial growth factor, can stimulate cell proliferation, matrix production, and angiogenesis at the site of injury [27,28].

By enhancing the natural wound healing cascade, PRP injections may help regenerate and repair the damaged plantar fascia tissue more effectively than corticosteroid injections, which only provide temporary anti-inflammatory effects but do not address the underlying degenerative changes in the plantar fascia. This regenerative capability of PRP likely underlies its superior long-term outcomes for reducing pain and improving function in patients with chronic plantar fasciitis [29,30].

Our findings are consistent with several previous studies that have reported favorable outcomes with PRP injections in the management of plantar fasciitis [18-23]. The long-term benefits observed in the PRP group suggest that this treatment modality may be particularly useful for patients with chronic or recalcitrant plantar fasciitis who have not responded to conservative management.

The strengths of this study include its prospective randomized design and the use of validated outcome measures. Limitations include the relatively small sample size, short follow-up duration, and use of only subjective patient-related outcome measures. This study did not include a subgroup analysis based on the age, gender, and BMI of the patients. Further studies with larger cohorts, longer-term follow-up, objective patient-related outcomes, and subgroup analysis are warranted to confirm the sustained efficacy of PRP in this patient population.

## Conclusions

Local injection of autologous PRP is more effective than corticosteroid injection in providing long-term pain relief and functional improvement in patients with chronic plantar fasciitis. Platelet-rich plasma should be considered as a viable treatment option. Especially for individuals with persistent or recalcitrant symptoms that have not responded to conservative management.
